# Overcoming resistance of *Candida albicans* using photodynamic inactivation

**DOI:** 10.1111/php.14108

**Published:** 2025-05-15

**Authors:** Gabriela Gomes Guimarães, Jennifer Soares, Anna Luiza Resende, Isabella Gonçalves, Kate Blanco, Vanderlei Bagnato

**Affiliations:** ^1^ PPGBiotec Federal University of São Carlos Sao Carlos Brazil; ^2^ São Carlos Institute of Physics, University of Sao Paulo Sao Carlos Brazil; ^3^ Biomedical Engineering Texas A&M University College Station Texas USA

**Keywords:** antifungal resistance, *Candida albicans*, combination therapy, photodynamic inactivation

## Abstract

The increasing resistance to conventional antifungal agents, such as Amphotericin B (AmB), has led to a growing demand for alternative therapeutic approaches for *Candida albicans*, an opportunistic fungal pathogen responsible for infections in immunocompromised patients. This study aimed to evaluate the effectiveness of photodynamic inactivation (PDI) in combination with AmB for controlling *C. albicans* growth, particularly in its yeast and hyphal forms, and to assess the impact of multiple PDI doses. *C. albicans* (ATCC 90028) was cultured in yeast and hyphal suspensions that were adjusted to 10^8^ CFU/mL and treated with AmB at varying concentrations (0.065–1.04 μg/mL), with and without PDI. PDI was performed using the photosensitizer curcumin (2.5 μM), activated by a 450 nm LED light source at a fluence of 15 J/cm^2^. The effect of single and repeated PDI doses was evaluated in the fungal biomolecules, which were assessed using Fourier transform infrared (FTIR) spectroscopy. Optical density (OD) measurements quantified fungal growth reduction at 540 nm. The combination of AmB and PDI significantly reduced *C. albicans* growth, achieving a 75% reduction in the yeast form and an 87.5% reduction in the hyphal form. Two doses of PDI further enhanced antifungal efficacy, particularly against hyphae, which exhibited higher sensitivity to treatment. These findings suggest that PDI enhances the antifungal action of AmB, particularly in more resistant *C. albicans* forms such as hyphae and biofilms. The observed synergistic effect supports the potential use of PDI as an effective strategy to combat antifungal resistance in clinical applications.

## Graphical abstract

The rising resistance of *Candida albicans* to conventional antifungals, like Amphotericin B (AmB), highlights the need for alternative treatments. This study evaluated the antifungal effectiveness of combining AmB with photodynamic inactivation (PDI) using curcumin as a photosensitizer. The combination significantly reduced fungal growth, especially in the hyphal form, which is more resistant. Two PDI doses enhanced the effect even further. Results suggest that PDI boosts the action of AmB and could help overcome antifungal resistance, offering a promising strategy for treatment.

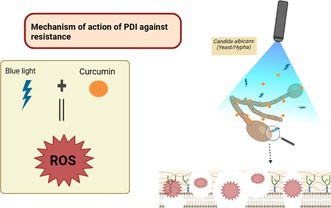

**Abbreviations**
CFU: colony forming unit
LED: light emitting diode
PBS: phosphate buffered saline
PDI: photodynamic inactivation
ROS: reactive oxygen species
RPM: revolutions per minute

## INTRODUCTION

Invasive fungal infections, particularly candidemia caused by *Candida albicans*, represent a significant challenge in immunocompromised patients, with mortality rates ranging from 36% to 63%. The ability of *C. albicans* to switch between morphological forms, such as yeast and hyphae, contributes to its virulence and resistance to antifungal treatments.^1^ Additionally, the COVID‐19 pandemic has exacerbated fungal infections in critically ill patients. The immunosuppressive effects of the virus, combined with corticosteroid use and mechanical ventilation, have created a favorable environment for opportunistic infections, including candidemia and aspergillosis.^2^

The morphological plasticity of *C. albicans* is a key factor in its pathogenicity. Yeast forms are more suited for bloodstream dissemination, whereas hyphal forms are more invasive and crucial for tissue penetration. Host tissue contact often triggers the transition from yeast to hyphae, where the hyphal form facilitates invasion and persistence. This morphological shift also enables immune evasion, as immune cells may respond differently to yeast and hyphal forms.^3^ Both forms contribute to biofilm formation, with hyphae playing a critical role in the adhesion and invasion of medical device surfaces. The protective environment within biofilms creates a microenvironment that reduces antifungal penetration, allowing *C. albicans* to persist despite antifungal treatment. The coexistence of yeast and hyphal forms within biofilms complicates fungal infection eradication.^4^

Biofilms, commonly found on mucosal surfaces and implanted medical devices, represent a significant challenge in treating *C. albicans* infections. Within biofilms, *C. albicans* exists in a dynamic equilibrium between its yeast and hyphal forms, contributing to biofilm pathogenicity and persistence. The hyphal form, which is more invasive and resistant to antifungal therapies, plays a central role in biofilm formation.^5^ Recent studies have suggested that PDI, particularly when combined with antifungal agents, enhances *C. albicans* biofilm treatment by targeting both yeast and hyphal forms while overcoming biofilm‐associated resistance mechanisms.^6^, ^7^ Among potential alternative therapies, PDI has emerged as a promising antimicrobial strategy.

Resistance to antifungal drugs, particularly azoles and amphotericin B, has become a growing concern in treating *C. albicans* infections. Several factors contribute to this resistance, including morphological plasticity (yeast and hyphae), biofilm formation, and cellular modifications. Antifungal resistance significantly limits treatment options in immunocompromised patients, particularly for systemic infections such as candidemia.^8^

A significant advantage of PDI is its ability to circumvent conventional resistance mechanisms, including biofilm formation, a key contributor to *C. albicans* persistence in infections.^9^ Moreover, when combined with conventional antifungal agents, PDI enhances drug efficacy by reducing the minimum inhibitory concentration (MIC) required for fungal inhibition, promoting synergy and improving treatment outcomes.^10^ This study aims to investigate the effectiveness of PDI as an innovative therapeutic approach for sensitizing *C. albicans* resistant to amphotericin B. Specifically, it compares the response of yeast and hyphal forms to PDI to assess its potential as an adjunct therapy in overcoming antifungal resistance. By exploring the differential responses of *C. albicans* morphologies to PDI, this study provides insights into the clinical implications of combining PDI with conventional antifungal treatments such as amphotericin B.

## MATERIALS AND METHODS

### Photosensitizer and light

This study used synthetic curcumin (EMI Pharma, São Carlos, SP, Brazil) as the photosensitizer. Before each experiment, a curcumin stock solution was prepared. Initially, 0.06 g of curcumin was weighed into a 10 mL volumetric flask and dissolved in 10 mL of dimethyl sulfoxide (DMSO) to prepare a stock solution at a final concentration of 16 mM. The working solution was obtained by diluting the stock solution in sterile saline to a final concentration of 2.5 μM in a Falcon tube, resulting in a final DMSO concentration of 0.1%. The Technical Support Laboratory at the São Carlos Institute of Physics, University of São Paulo (USP), developed a handpiece containing a blue light‐emitting diode (450 nm, Biotable).

### Microorganism

The microorganism used in this study was *C. albicans* (ATCC 90028). The culture was stored in microtubes containing Tryptic Soy Broth (TSB) with 25% glycerol and frozen at −20°C. Before the experiment, the yeast was reactivated on Sabouraud Dextrose Agar (SDA) and incubated at 37°C for 48 h. The colonies were suspended in phosphate‐buffered saline (PBS) for PDI assays. For antifungal treatments, the colonies were suspended in TSB. The inoculum was adjusted to a final concentration of 10^8^ CFU/mL based on optical density (OD) measurements at 540 nm (Cary UV–Vis 50, Varian). Biofilms were developed for experiments involving hyphal forms. To achieve this, 1 mL of the adjusted suspension was transferred to 24‐well polystyrene culture plates and incubated at 37°C in a shaking incubator (75 rpm) for 90 min to allow fungal cell adhesion. After this period, the suspension was removed, and the wells were washed twice with PBS to eliminate non‐adherent cells.

Next, 1 mL of TSB was added to each well, and the plates were incubated under agitation (75 rpm) at 37°C for 24 h to allow biofilm formation. Following this incubation, the suspension was discarded, the biofilms were washed twice with PBS, and the adhered biofilm was detached from the wall surfaces. The detached cells were suspended in PBS for PDI assays, whereas the detached cells were suspended in TSB for antifungal treatments. To ensure experimental reproducibility, all procedures were performed under strictly controlled incubation times and conditions.

### Minimum inhibitory concentration

The experiments were conducted with adaptations according to the recommendations of the BrCAST (Brazilian Committee on Antimicrobial Susceptibility Testing). Then, the fungal inoculum was added to each well, adjusting the final volume to 100 μL. Positive controls for fungal growth and negative controls containing only the culture medium were also included. The plate was incubated at 37°C for 24 h. After incubation, the OD of the samples was measured using a UV–VIS microplate reader. The minimum inhibitory concentration (MIC) was defined as the lowest concentration of the antifungal agent capable of inhibiting visible fungal growth, as indicated by the OD. These assays were conducted for yeast and biofilm forms to evaluate antifungal effects under different fungal growth conditions. The Equation 1 for calculating the percentage reduction is:

(1) $$\text{Percent reduction}=\left(\frac{{\mathrm{MIC}}_{\text{control}}-{\mathrm{MIC}}_{\text{treated}}}{{\mathrm{MIC}}_{\text{control}}}\right)\times 100$$

where MIC_control_ represents the minimum inhibitory concentration of the control group (without treatment), serving as a baseline reference for fungal growth and MIC_treated_ corresponds to the minimum inhibitory concentration measured after treatment, reflecting the effectiveness of the antifungal intervention.

By subtracting the final value from the initial value, we determine the growth reduction caused by the treatment. Dividing this difference by the initial value and multiplying by 100 gives us the reduction percentage. This calculation provides a precise and quantitative measure of how effective the treatment is in inhibiting fungal growth.

### Photodynamic inactivation

The colonies were suspended in phosphate‐buffered saline (PBS) for PDI assays and adjusted to a density of 10^8^ CFU/mL. Yeast cells were incubated with the photosensitizer for 20 min. Following incubation, the PDI treatment (yeast + PS + light) was performed, with the group exposed to a light dose of 15 J/cm^2^ using a 450 nm LED device (Biotable).

After the PDI application, the cells were collected, transferred to Falcon tubes, and centrifuged at 1790 × ***g*** for 5 min. The pellet was resuspended in saline solution. Part of the samples was incubated again with curcumin at 2.5 μM for 20 min, while another part was suspended in TSB for minimum inhibitory concentration (MIC) assays. This procedure was repeated once more, totaling two PDI cycles. For hyphal tests, biofilms were incubated with the photosensitizer for 20 min. Following incubation, PDI treatment (biofilm + PS + light) was applied, exposing the samples to a light dose of 15 J/cm^2^ using the 450 nm LED device. After treatment, a pipette tip detached the biofilm from the plate surface. The detached cells were collected, transferred to Falcon tubes, centrifuged at 4000 rpm for 5 min, and resuspended in saline solution. As with the yeast protocol, a portion of the samples was incubated again with curcumin at 2.5 μM for 20 min, while the remaining portion was suspended in TSB for MIC assays. This procedure was also repeated once, totaling two PDI cycles. Following treatment, the plates were incubated at 37°C for 24 h. The OD of the samples was measured using a UV–VIS microplate reader. The minimum inhibitory concentration (MIC) was defined as the lowest antifungal concentration capable of inhibiting visible fungal growth, as determined by OD measurements. Table 1 summarizes the treatment conditions, including incubation times and light doses applied in the PDI groups.

**TABLE 1:** Photoinactivation efficiency of Candida albicans under different treatment conditions.

Group	Treatment	Composition	Incubation time	Light dose (J/cm^2^)	Objective
Control group	No treatment	–	–	–	Assess basal growth of *Candida albicans*
PDI (yeast)	PDI + Curcumin + Light	Yeast + PDI (Curcumin 2.5 μM + Blue LED Light)	20 min (incubation with curcumin)	15 J/cm^2^	Assess PDI effect on yeast
Antifungal (MIC)	Antifungal (Amphotericin B) + TSB Medium	*C. albicans* + Antifungal	24 h	–	Assess antifungal effect
PDI + Antifungal + Yeast	PDI + Antifungal + yeast	*C. albicans* + PDI (Curcumin 2.5 μM) + Antifungal	20 min (incubation with curcumin)	15 J/cm^2^	Assess the effect of PDI + antifungal on yeast
Hypha—PDI (Hyphae)	PDI + Curcumin + Light	Hyphae of *C. albicans* + PDI (Curcumin 2.5 μM + Blue LED Light)	20 min (incubation with curcumin)	15 J/cm^2^	Assess PDI effect on hyphae
Antifungal (MIC) (Hyphae)	Antifungal (Amphotericin B) PDI + Hyphae + Antifungal	Hyphae of *C. albicans* + Antifungal	24 h	–	Assess the antifungal effect on hyphae
PDI + Antifungal + Hyphae	PDI + Antifungal + Hyphae	Hyphae of *C. albicans* + PDI (Curcumin 2.5 μM) + Antifungal	20 min (incubation with curcumin)	15 J/cm^2^	Assess the effect of PDI + antifungal on hyphae
Control hyphae	No treatment	Hyphae of *C. albicans*	–	–	Assess hyphae growth

### Growth curves

The microbial growth curve for the PDI combination was analyzed in *C. albicans*. The PDI protocols used were the same as those applied in successive doses. Initially, PDI was performed to evaluate its impact on the growth rate over 16 h. During this period, OD was measured at 540 nm for each treatment group, including monotherapies and the control group, to assess the growth curves of the respective treatments.

The experiments were conducted in 96‐well plates, with the addition of *C. albicans* without PDI to establish baseline growth. Subsequently, the effect of PDI alone on fungal growth was evaluated. In addition, antifungal doses equivalent to the MIC were administered to analyze the yeast's growth behavior in response to the antifungal and combined PDI treatment.

### FTIR spectroscopy, data processing, and analysis

After the final centrifugation, the pellet was resuspended in a small volume of distilled water to obtain a concentrated suspension. Due to the FTIR equipment's sensitivity to water, the suspension was left to rest in a controlled environment at room temperature until most of the water had evaporated. This procedure was performed to minimize water interference in the FTIR spectra.^11^ Subsequently, the analysis was conducted using an Attenuated Total Reflection Fourier Transform Infrared (ATR‐FTIR) spectrometer. A dry fungal sample was evenly distributed on the surface of the equipment's crystal, ensuring efficient sample transfer for analysis. The ATR‐FTIR crystal was composed of either diamond or ZnSe.^12^

The dry sample was scanned 250 times using the ATR‐FTIR spectrometer, and the result was obtained as the average of these measurements to ensure data accuracy. The FTIR spectrum was recorded in the range of 4000 to 500 cm^−1^, covering the bands corresponding to functional groups of specific interest, including carbohydrates, lipids, proteins, and nucleic acids.^13^ Measurements were performed on four samples, with multiple spectra acquired for each sample to ensure reproducibility. The resulting spectra were subjected to second derivative analysis, followed by normalization using the min–max method, allowing effective comparisons between the analyzed samples.

### Statistical analysis

The experiments were conducted in biological triplicates (*N* = 9), and the normality of the data was assessed using the Shapiro–Wilk test. The results indicated that the data followed a normal distribution (*p* > 0.05 for all groups). Given that the normality assumption was met, statistical analyses were conducted using one‐way analysis of variance (ANOVA) to compare the groups, followed by the Tukey test for multiple comparisons (*α* = 0.05). In addition, to evaluate the interaction between photodynamic inactivation (PDI) and Amphotericin B, we applied the Bliss independence model. This mathematical approach allowed us to determine whether the combined effect of the treatments was synergistic, additive, or antagonistic, based on the predicted versus observed viability reduction in treated samples. The Bliss analysis was used to support the interpretation of potential therapeutic advantages of the combined treatment protocol.

## RESULTS AND DISCUSSION

**FIGURE 1:**
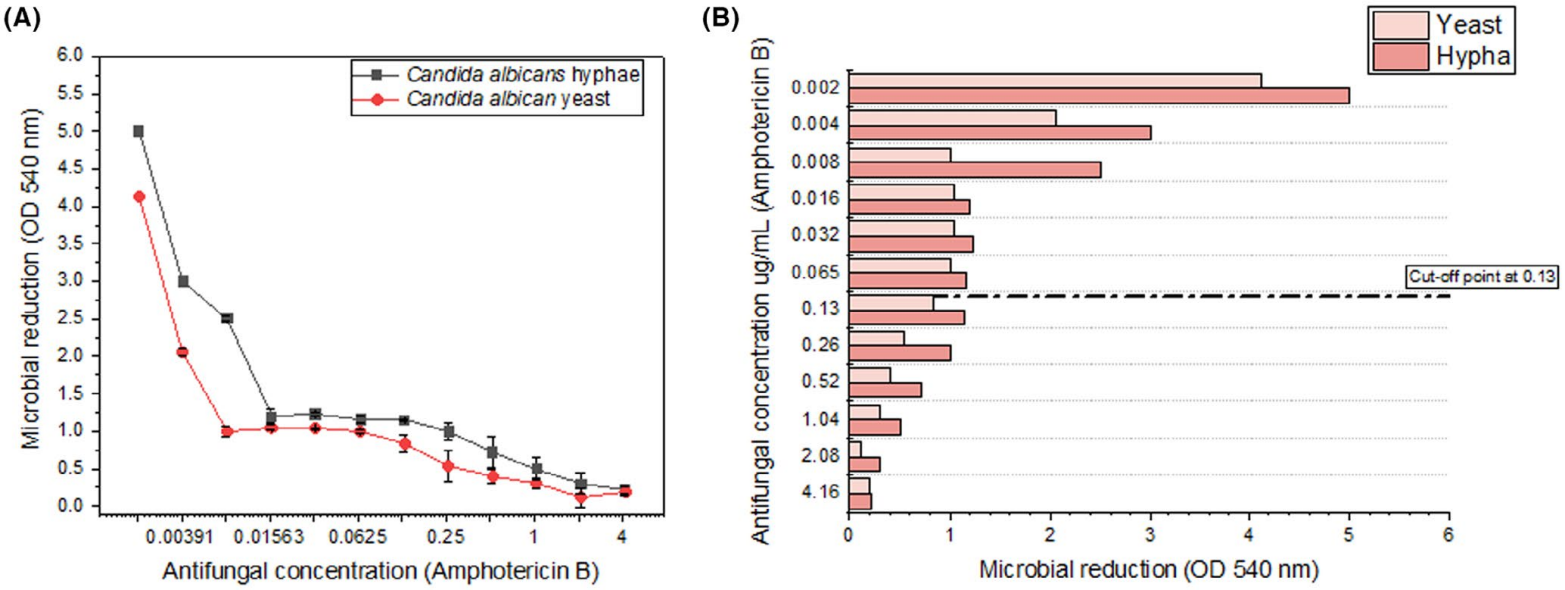
(A) Effect of increasing concentrations of AmB on the growth of *Candida albicans* in both yeast and hyphal forms. Growth inhibition was assessed by measuring optical density (OD) at 540 nm. As the concentration of AmB increased, both yeast and hyphal growth were significantly reduced. The most effective concentration for inhibiting growth was 0.26 μg/mL for yeast cells, while for hyphal cells, it was 1 μg/mL. A more pronounced reduction in OD was observed in yeast cells, which showed greater sensitivity to AmB than hyphal cells. These results suggest that the yeast forms of *Candida albicans* are more susceptible to AmB than the hyphal forms, and higher concentrations of AmB are required to inhibit hyphal growth. (B) The graph shows microbial reduction (OD 540 nm) as a function of Amphotericin B concentration, with a focus on the cutoff point at 0.13 μg/mL. At this concentration, yeast cells exhibit a more significant reduction in growth compared with hyphal cells, highlighting their greater sensitivity to AmB. The Minimum Inhibitory Concentration (MIC) for yeast cells is 0.26 μg/mL, while for hyphal cells, it is 1.04 μg/mL, demonstrating that hyphae require approximately four times the concentration of AmB to achieve similar levels of inhibition. These results underscore the difference in sensitivity between the two morphological forms of Candida albicans, with yeast cells being more susceptible to AmB even at sub‐MIC.

**FIGURE 2:**
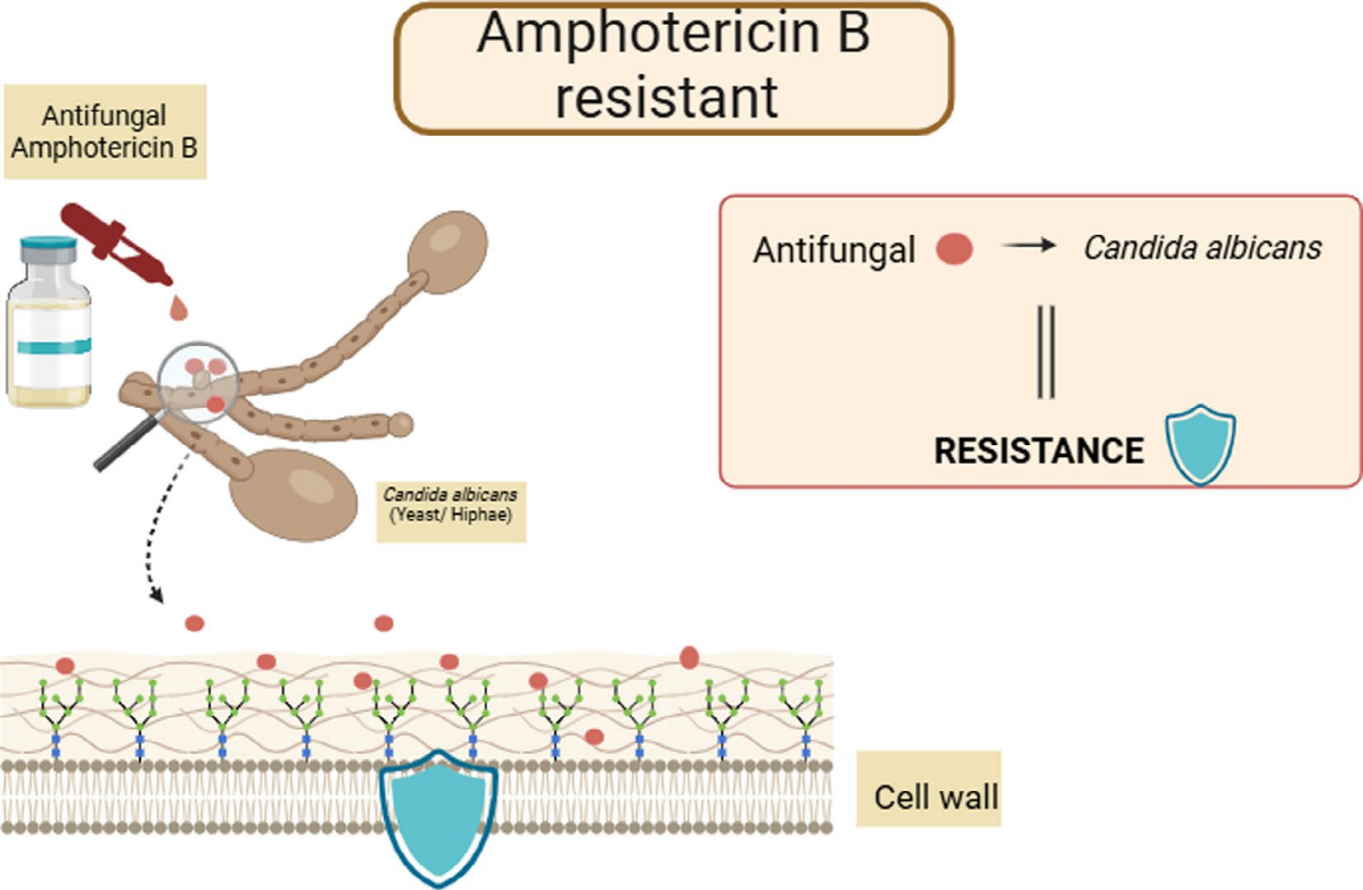
Representation of *Candida albicans* resistance to amphotericin B. The illustration shows the fungus's yeast and hyphal forms, highlighting the antifungal (amphotericin B) application and its difficulty in penetrating the cell wall due to resistance. The blue shield symbolizes the resistance barrier, indicating the antifungal's reduced efficacy against *Candida albicans*.

The effect of increasing concentrations of AmB on *C. albicans* growth was evaluated for both yeast and hyphal forms. Growth inhibition was quantified by measuring OD at 540 nm after treatment. Figure 1A,B show a dose‐dependent response to AmB, with a significant reduction in fungal growth in both morphological forms. Notably, yeast cells exhibited greater sensitivity to AmB, as evidenced by a more pronounced decline in OD values at lower concentrations, as shown in Figure 2A. Figure 2B demonstrates that hyphal cells required a concentration four times higher than the yeast form to experience significant growth inhibition, reflecting their higher resistance to the antifungal. The minimum inhibitory concentration (MIC) for *C. albicans* was assessed by evaluating the percentage of survival, which was measured by optical density (OD) at 540 nm. The MIC for the yeast form was found to be 0.26 μg/mL, and for the hyphal form, the MIC was higher, requiring higher antifungal doses to inhibit growth, as shown in both figures. The resistance cutoff point for AmB was established at 0.13 μg/mL, meaning that both the yeast and hyphal forms exhibit resistance to the antifungal agent at concentrations below this threshold.

### Effect of AmB on the growth of *Candida albicans* yeast and hyphae

**FIGURE 3:**
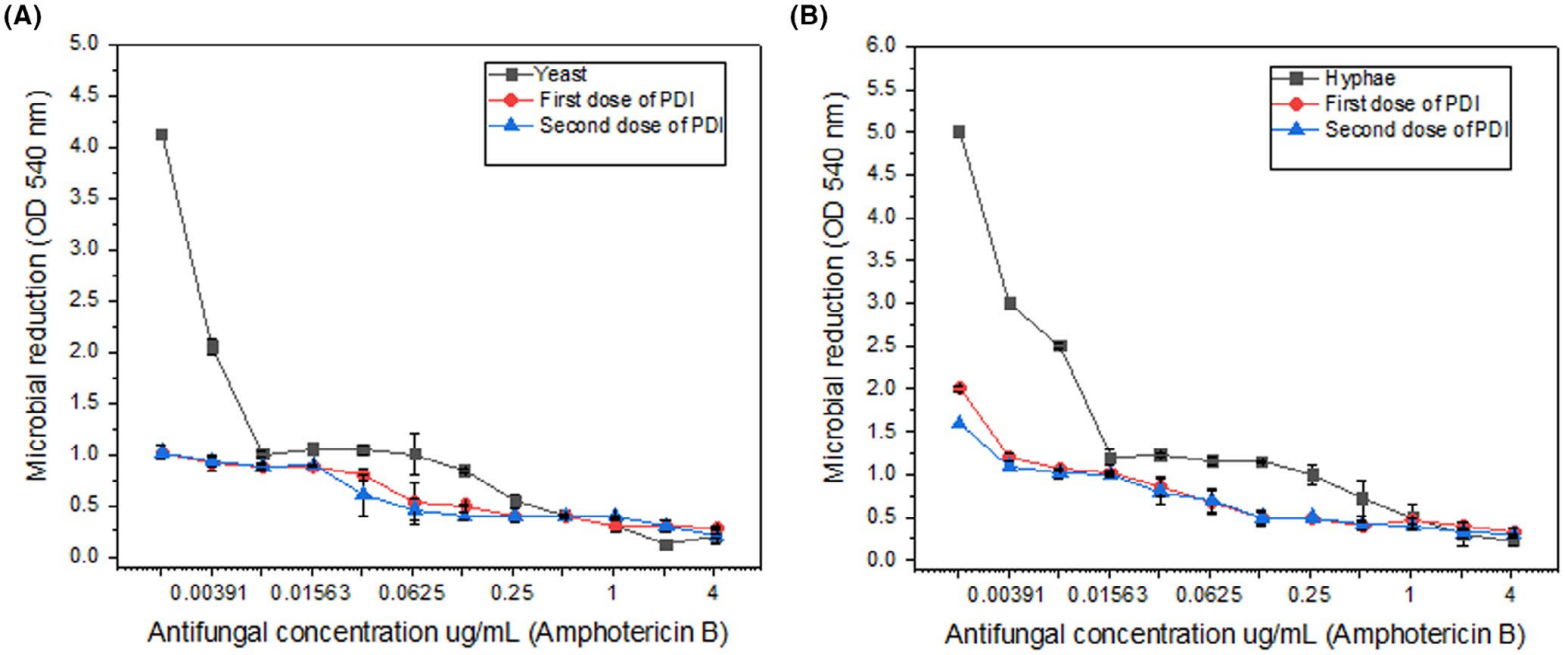
Effect of increasing concentrations of AmB combined with PDI on the growth of *Candida albicans* in its yeast and hyphal forms. OD measurements were used to assess fungal growth. The data demonstrates that the growth of both forms is significantly inhibited by AmB, with a more pronounced effect observed at higher concentrations. The addition of PDI, particularly with two doses, enhances the inhibitory effect, especially in hyphal cells. Hyphal forms exhibit greater sensitivity to treatment compared with yeast cells, highlighting the differential response of *C. albicans* to AmB and PDI in a dose‐dependent manner.

The results shown in Figure 2 demonstrate a dose‐dependent antifungal effect of AmB on *C. albicans*, as evidenced by the progressive reduction in the growth of the yeast form with increasing concentrations of the compound. This inhibition is more pronounced at higher concentrations, suggesting that AmB directly interferes with cellular proliferation mechanisms. Similarly, the growth of the hyphal form of *C. albicans* also exhibited a significant reduction with increasing AmB concentrations. Notably, inhibition occurred at lower concentrations when compared to the yeast form, indicating that hyphal cells are more sensitive to AmB than their yeast counterparts. This differential susceptibility may be attributed to structural and physiological variations between these morphological states. However, as illustrated in Figure 3, resistance mechanisms in *C. albicans* can hinder the antifungal efficacy of AmB, with the fungal cell wall acting as a protective barrier against the drug.

Data analysis identified that the most effective concentration for inhibiting the growth of *C. albicans* varied according to the yeast's morphological form. For the hyphal form, the MIC was 1.04 μg/mL, indicating that this form exhibits greater resistance to the antifungal, requiring higher doses of AmB for effective growth inhibition. On the other hand, the yeast form had an MIC of 0.26 μg/mL, indicating that it is more susceptible to AmB at lower concentrations. The resistance cutoff point was determined to be 0.13 μg/mL, indicating that both yeast and hyphal forms are resistant to AmB at concentrations below this threshold. Growth inhibition was quantified by measuring OD at 540 nm, and a significant reduction in OD values was observed, further supporting these findings. These results suggest that the morphology of *C. albicans* influences its sensitivity to antifungal treatment, with hyphal forms demonstrating greater resistance and requiring higher concentrations of the antifungal to achieve the same level of growth inhibition compared to the yeast forms.

### Effect of AmB combined with PDI on *C. albicans* growth

Figure 3 illustrates the effect of increasing concentrations of AmB combined with PDI on the growth of *C. albicans* in yeast and hyphal forms. Fungal growth was assessed through OD measurements at 540 nm. The data clearly show that the combination of AmB and PDI significantly enhances growth inhibition in both morphologies. In this study, we evaluated the activity or growth of hyphae and yeast of Candida by analyzing the area under the curve. The results showed significant differences between the two morphological forms. The area for hyphae was 1.8051, while the area for yeast was 1.07575. In percentage terms, the area of yeast corresponds to 59.6% of the area of hyphae, indicating that yeast exhibited approximately 40% less activity or growth compared with hyphae. On the contrary, the area of hyphae is 67.8% larger than the area of yeast, reinforcing the idea that hyphae have a more robust or resistant response to the tested conditions. This difference may be related to factors such as the greater resistance of hyphae to treatments or a faster growth capacity. The analysis of these data suggests that hyphae are more active or resistant compared with yeast, which may have important implications for the development of therapeutic strategies targeting fungal infections caused by *Candida*. The analysis of the area under the curve revealed significant differences between the groups. In the control group, the area was 1.07575. After one dose of PDI, the area increased to 1.33671, representing a 24.3% increase compared with the control. With two doses of PDI, the area slightly decreased to 1.31015 but remained 21.8% higher than the control. These results suggest that PDI increased the dispersion of yeast growth while reducing the peak growth. Furthermore, the second dose of PDI did not show a significantly different effect from the first, indicating that a single application may be sufficient to achieve the desired effect. Analysis of the area under the curve revealed significant differences between the groups. In the control group, the area was 1.8051. After one dose of PDI, the area decreased to 1.69644, corresponding to a 6.0% reduction compared with the control. With two doses of PDI, the reduction was even more pronounced, reaching 1.50968, which represented a 16.4% decrease compared with the control. The combination of photodynamic inactivation (PDI) with Amphotericin B was evaluated using the Bliss independence model to determine whether the observed effects were synergistic, additive, or antagonistic. The results demonstrated that PDI significantly reduced the viability of both morphological forms of *C. albicans*, with a greater impact observed in the filamentous (hyphal) form.

For the yeast form, PDI alone reduced cell viability by 87.5%, while Amphotericin B alone achieved a 75% reduction. According to the Bliss model, a purely additive effect would predict a reduction of 96.88% in cell viability. However, the observed effect exceeded this value, indicating a synergistic interaction between PDI and Amphotericin B, thereby enhancing the antifungal efficacy against the yeast form.

In the hyphal form, an even more pronounced effect was observed. While Amphotericin B alone required a concentration of 1.04 μg/mL to inhibit fungal growth, this requirement was reduced to 0.13 μg/mL when combined with PDI–representing an 8‐fold decrease in the minimum inhibitory concentration (MIC). This high sensitivity of the hyphal form to the combined treatment further supports the existence of a synergistic effect. These findings highlight the potential of PDI as an adjuvant therapy to Amphotericin B, enabling lower drug dosages to be used, which may help minimize side effects and improve treatment efficacy, especially against more resistant fungal forms. Overall, the results reinforce the role of photodynamic inactivation as a promising strategy to overcome antifungal resistance and enhance conventional treatment protocols for *C. albicans* infections.

These results indicated that PDI was effective in inhibiting hyphal growth, with a dose‐dependent effect observed after multiple applications. Thus, our findings reinforced the potential of PDI as a promising therapeutic strategy for controlling fungal infections involving *Candida* hyphae.

The inhibitory effect is more pronounced at higher AmB concentrations, with PDI further potentiating fungal suppression. Notably, applying two PDI doses enhances the antifungal impact, leading to a more substantial reduction in OD values. This suggests that when used with AmB, PDI may contribute to increased fungal susceptibility, reinforcing its potential as an adjunctive therapy.

A comparison between yeast and hyphal forms revealed differential responses to treatment. Hyphae demonstrated heightened sensitivity to AmB and PDI, exhibiting a greater reduction in growth at lower concentrations than yeast cells. This finding suggests that the morphological transition of *C. albicans* influences its susceptibility to treatment, highlighting the importance of targeting hyphal structures in antifungal strategies. At concentrations around 0.26 μg/mL, the combination of PDI—particularly with two doses—significantly enhanced the inhibitory effect of AmB on yeast growth. This concentration emerged as the most effective for inducing fungal inhibition, as reflected in the sharp OD decrease in graphical analyses. In the control group, *C. albicans* hyphae without PDI treatment exhibited a significant reduction in growth with increasing concentrations of Amphotericin B, confirming its antifungal efficacy. However, the effect was less pronounced in yeast cells, emphasizing the differential susceptibility between the two morphological forms.

Applying a single dose of PDI led to a notable reduction in both yeast and hyphal growth. However, the inhibition was more pronounced in yeast cells, suggesting that PDI may be more effective against yeast than hyphal forms. The first dose of PDI complemented the antifungal action of Amphotericin B, enhancing its inhibitory effect on both morphological forms. Nevertheless, residual fungal growth persisted, particularly in hyphae, indicating that a single dose may not be sufficient for complete eradication. By contrast, a second PDI dose resulted in a significantly greater OD reduction, particularly in hyphal forms. This supports the hypothesis that repeated PDI applications are more effective at suppressing hyphal growth, which is often more resistant due to its structural complexity and biofilm formation potential. The second PDI dose further enhanced AmB activity, resulting in a substantial reduction in fungal viability. These findings suggest that multiple PDI doses could improve treatment efficacy, particularly when combined with conventional antifungal agents. A comparative analysis between yeast and hyphal cells indicates that hyphal forms exhibit higher resistance to treatment with antifungal agents alone. This is consistent with the literature, which suggests that hyphal structures confer increased resilience due to their complex morphology and biofilm‐forming ability. However, the combination of PDI with AmB significantly enhances antifungal activity, likely through ROS‐mediated oxidative damage, making PDI a promising adjuvant therapy.

### Effect of AmB and PDI on the growth of *Candida albicans* yeast and hyphae

The study investigates the impact of AmB in combination with photodynamic therapy (PDI) on the growth of *C. albicans*, both in its yeast and hyphal forms. The results demonstrate that *C. albicans* growth in the yeast form is inhibited by AmB (Figure 5), with more pronounced inhibition observed at higher concentrations. The results indicate a significant antifungal effect of AmB, particularly at higher concentrations, where yeast growth is substantially inhibited. Adding PDI enhances this inhibitory effect, especially with multiple doses, further reducing fungal viability. Notably, a single dose of PDI already increases the antifungal effect compared with AmB alone. However, the group receiving two doses of PDI exhibited the most substantial reduction in growth, even at lower AmB concentrations. Statistical analyses were performed to compare the different experimental groups. To verify the existence of significant differences between the groups, a one‐way ANOVA was performed. Initially, descriptive statistics were calculated for the three conditions analyzed: yeast Control, PDI1, and PDI2. The means and standard deviations were, respectively, 0.65376 ± 0.03436 (yeast Control), 0.63217 ± 0.04293 (PDI1), and 0.54047 ± 0.0393 (PDI2). The assumption of normality of the data was assessed using the Shapiro–Wilk test, whose *p* values ranged from *p* = 0.13821 to *p* = 0.98028, indicating that there was no evidence to reject the normality of the data (*p* > 0.05 for all groups). Therefore, the data were considered normally distributed.

The ANOVA revealed a significant difference between the groups, suggesting that at least one of the groups had a significantly different mean from the others. To identify which groups differed from each other, Tukey's post‐hoc test was performed, which indicated a significant difference between PDI 2 and the other groups (*p* < 0.05), while PDI 1 and yeast Control did not show significant differences between them (p > 0.05). Therefore, the results suggest that the PDI 2 condition presented significantly lower values compared with the other groups (p < 0.05), while PDI 1 did not differ statistically from yeast Control (p > 0.05).

The descriptive statistics indicated that the hypha group had a mean of 0.70476 ± 0.09869, while the PDI 1 and PDI 2 groups had means of 0.55333 ± 0.04509 and 0.53141 ± 0.06078, respectively. The one‐way ANOVA revealed a significant difference between the groups (*p* = 0.049), suggesting that at least one of the groups differs significantly from the others. To identify where these differences occurred, Tukey's post‐hoc test was applied for multiple comparisons. The comparison between PDI 1 and hypha showed a mean difference of −0.15143, with *p* = 0.092. The comparison between PDI 2 and hypha showed a mean difference of −0.17335, with *p* = 0.057. The comparison between PDI 2 and PDI 1 showed a mean difference of −0.02192, with *p* = 0.927. None of these comparisons reached statistical significance at the 0.05 level, indicating that, although the ANOVA revealed a global significant difference, the specific pairwise comparisons were not statistically significant.

Additionally, the Shapiro–Wilk test was performed to assess the normality of the data. The results indicated that the data from all groups followed a normal distribution, with p values for each group as follows: hypha: *p* = 0.752, PDI 1: *p* = 0.878, and PDI 2: *p* = 0.408. These results confirmed the appropriateness of using parametric methods for the analyses.

**FIGURE 4:**
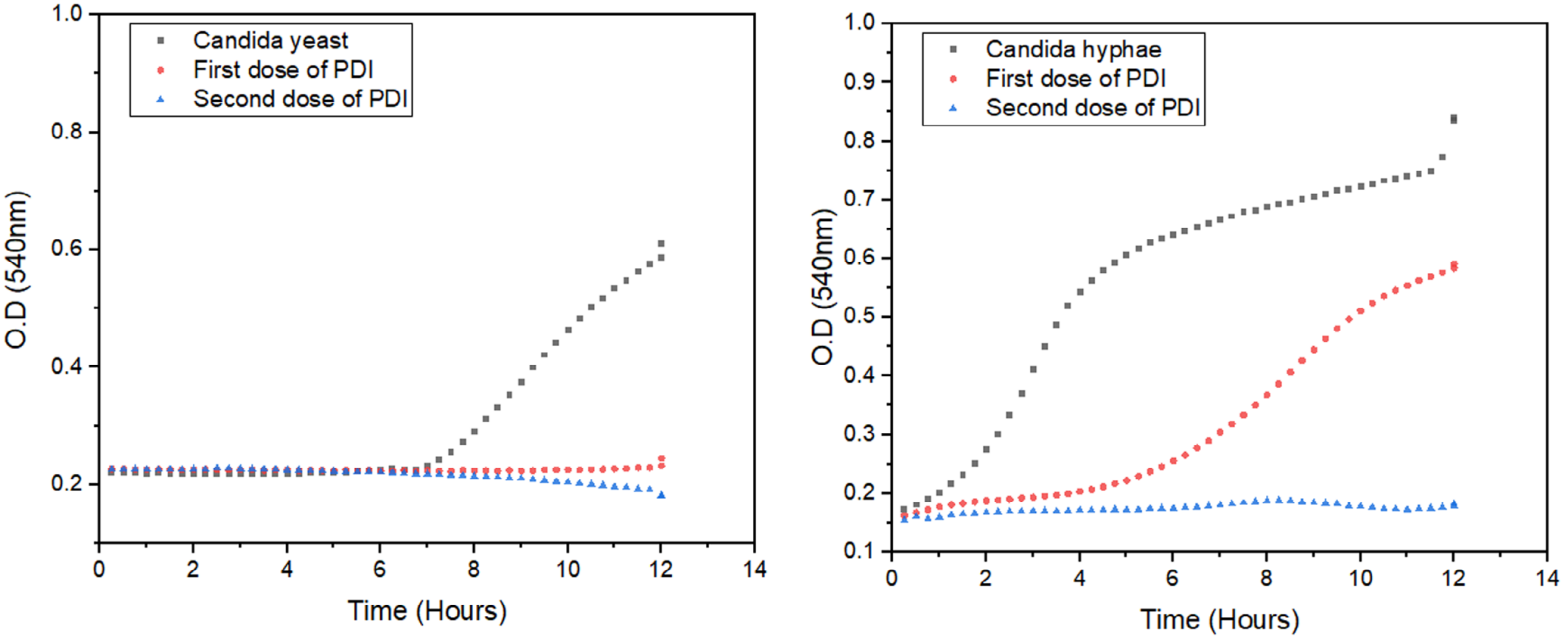
Growth curve analysis of *Candida albicans* yeast and hyphae treated with photodynamic inactivation (PDI). The control group (no PDI) exhibits the highest optical density, indicating robust yeast and hyphal growth. A single dose of PDI leads to a noticeable reduction in growth, while two doses result in an even greater decrease, demonstrating a dose‐dependent inhibitory effect. The cumulative impact of two doses suggests enhanced generation of reactive oxygen species (ROS), leading to increased cellular damage and suppression of both yeast proliferation and hyphal formation.

Figure 4 presents the growth curve analysis of *C. albicans* yeast and hyphae subjected to PDI treatment. *Candida* yeast without treatment, treated with a single dose of photodynamic inactivation (PDI), and treated with two doses of PDI. The control curve (black squares) exhibits exponential growth after approximately 8 h, indicating significant yeast proliferation. By contrast, the curve corresponding to the first dose of PDI (red circles) remains stable over time, with a slight increase towards the end of the experiment, suggesting considerable growth inhibition but without complete eradication. The curve for the second dose of PDI (blue triangles) remains even more stable and shows a slight decline, indicating a more pronounced inhibitory effect compared with the first dose. These findings demonstrate that PDI effectively suppressed yeast growth, with the first dose already promoting substantial inhibition and the second dose enhancing this effect.

The control group, which did not receive PDI, exhibited the highest OD, indicating robust hyphal growth, that is, at 10 h, the OD is 0.75. A single dose of PDI resulted in a noticeable reduction in OD, demonstrating its inhibitory effect, that is, at 10 h, the OD is 0.57. However, two doses of PDI led to a more pronounced decrease in fungal growth, underscoring the dose‐dependent nature of PDI in inhibiting hyphal formation, that is, at 10 h, the OD 0,16. The cumulative effect of two PDI applications suggests enhanced generation of reactive oxygen species (ROS), leading to more significant cellular damage and increased inhibition of hyphal growth. This is also backed up by the change in generation time, which for no PDI is approximately 2 h, while after the application of 1 cycle of PDI, it is approximately 5 h. In the case of the 2‐cycle growth, it is practically absent.

**FIGURE 5:**
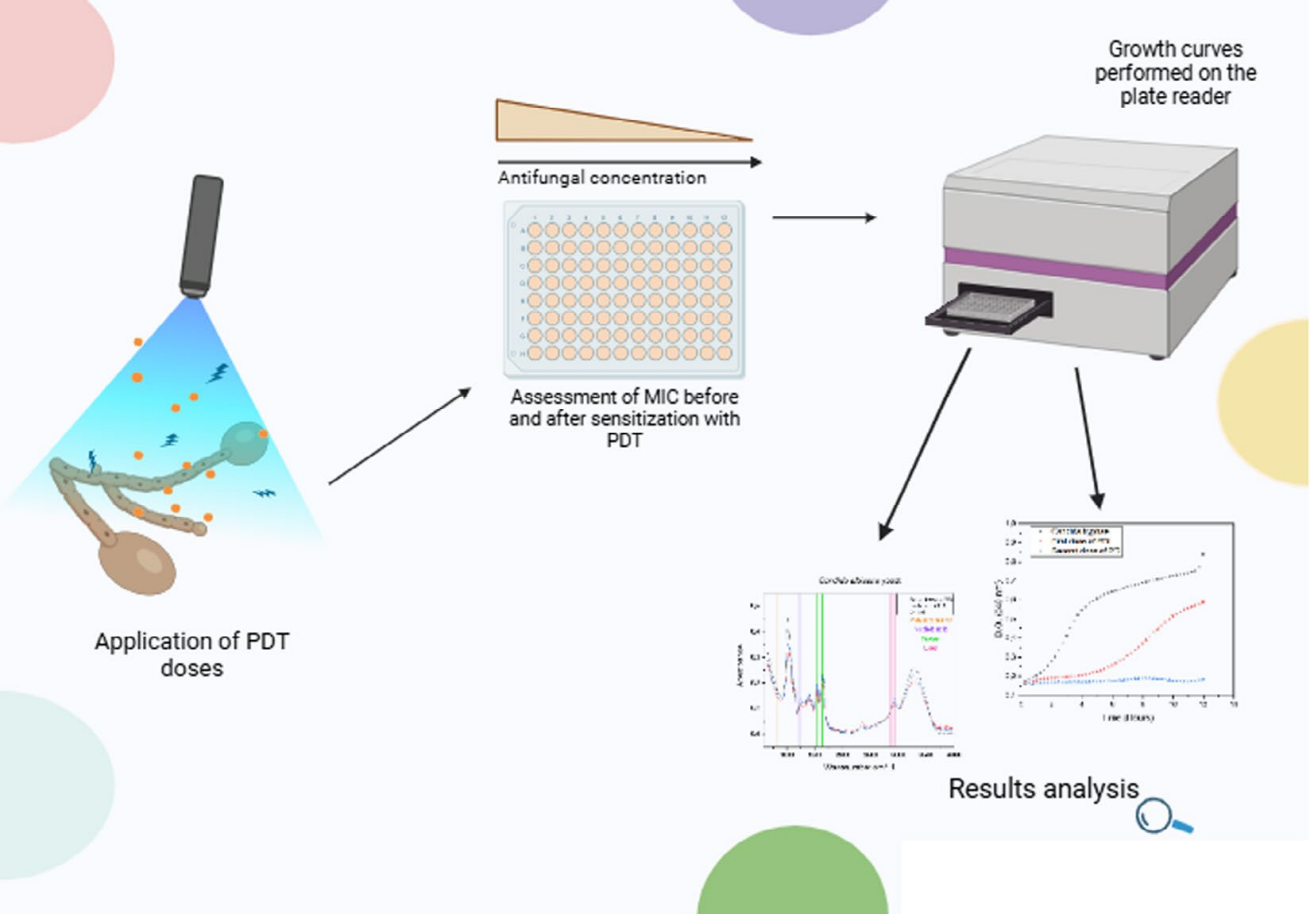
Representation of the experimental approach: *Candida albicans* cells are first subjected to PDI treatment, followed by exposure to different concentrations of amphotericin B (MIC assay).

Figure 5 illustrates the experiment performed with successive doses of PDI. After the doses, the sensitized *Candida* (both in its hyphal and yeast forms) is exposed to the MIC assay with different concentrations of amphotericin B. Subsequently, growth curves and FTIR analysis are conducted to assess the behavior of *Candida* exposed to PDI and its responses to successive doses in combination with varying concentrations of the antifungal amphotericin B.

### Effect of PDI on yeast and hypha Forms of *C. albicans*

Analysis of the Fourier Transform Infrared Spectroscopy (FTIR) graph, which presents normalized absorbance as a function of wave number (cm^−1^), reveals significant changes in the chemical and structural composition of yeast samples subjected to different treatments (control, PDI 1, and PDI 2). The FTIR technique identifies functional groups and chemical bonds in biological samples such as yeast. It can provide valuable insights into the effects of antimicrobial treatments such as photodynamic inactivation (PDI).

**FIGURE 6:**
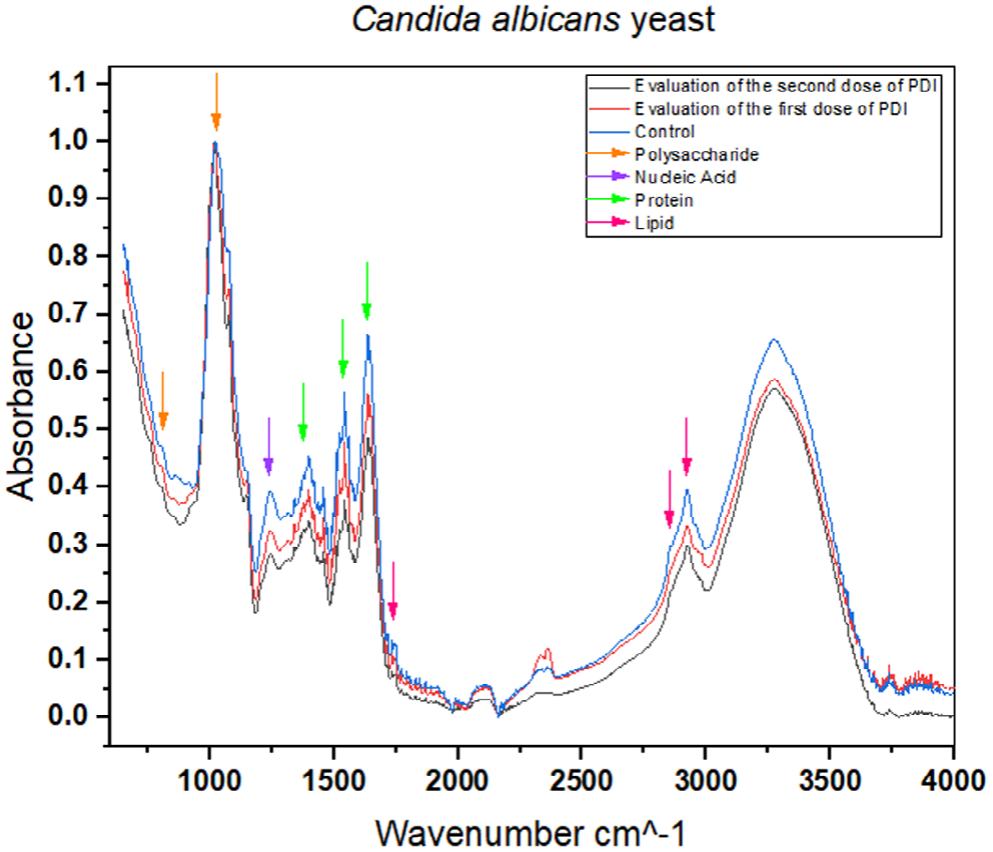
The graph shows the normalized absorbance as a function of wave number (cm^−1^) for yeast samples: Control (blue line), PDI1 (red line), and PDI2 (black line).

Figure 6 shows the regions analyzed in detail. In the spectral region from 1000 to 1500 cm^−1^, which corresponds to vibrations of C–O and C–C bonds, peaks associated with carbohydrates and cell wall polysaccharides are observed. Changes in this region, such as reductions in absorbance or peak shifts, may indicate damage to the yeast cell wall. Suppose the graph shows significant changes in this region for PDI 1 and PDI 2. This suggests that the treatments affect the yeast cell wall, possibly by generating reactive oxygen species (ROS).

**FIGURE 7:**
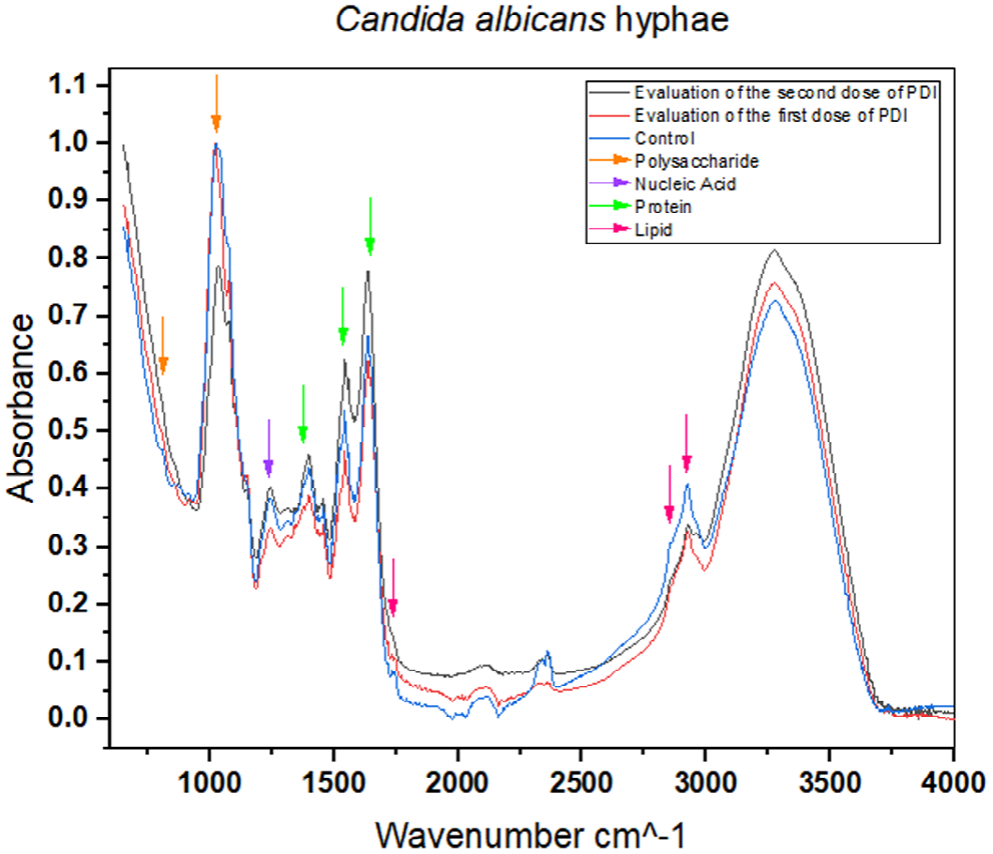
The graph shows the normalized absorbance as a function of wave number (cm^−1^) for hyphae simples: Control (blue line), PDI1 (red line), and PDI2 (black line).

In the region from 1500 to 1700 cm^−1^, which includes vibrations of C=O (carbonyl) and N–H (amide) bonds, peaks related to proteins and peptides are observed. Changes in this region, such as intensity or peak shifts, may indicate protein denaturation or aggregate formation. Figure 7 shows changes in this region for PDI 1 and PDI 2, which suggest that the treatments are damaging the yeast proteins and compromising their functionality.

The region from 2800 to 3000 cm^−1^, associated with C–H stretching vibrations, is relevant for analyzing lipids and fatty acids. Changes in this region, such as increases or decreases in absorbance, may indicate lipid peroxidation or changes in cell membrane fluidity. The graph shows changes in this region for PDI 1 and PDI 2, suggesting that the treatments are causing oxidative damage to membrane lipids.

Hydration and protein structure peaks are observed in the 3000 to 3500 cm^−1^ region, which includes stretching vibrations of O–H (hydroxyl) and N–H (amide). The graph shows changes in this region, which suggest that the treatments are affecting the structure and hydration of the yeast proteins.

FTIR spectroscopy revealed significant changes in essential components of the hypha, such as structural proteins, lipids of the plasma membrane, and polysaccharides of the cell wall (chitin and β‐glucans). The spectral differences between the control and the treated groups indicate that PDI progressively affects the integrity of the hypha, making it more susceptible to degradation.

Figure 7 shows the different regions analyzed. The spectral region between 3600 and 3000 cm^−1^ is associated with the vibrations of O–H and N–H bonds, reflecting the presence of polysaccharides and structural proteins. An increase in absorbance was observed after the first and second doses of PDI. This may indicate the formation of new intermolecular interactions or the modification of functional groups due to oxidative stress induced by photodynamic treatment.

Slight variations were detected between the control and the treated groups in the spectral range of 3000–2800 cm^−1^, corresponding to the C–H vibrations of aliphatic chains. This suggests modifications in lipid composition, indicating damage to the plasma membrane, a structure essential for cellular homeostasis and resistance to environmental stress. Previous studies have shown that PDI can cause lipid peroxidation, leading to the disorganization of the lipid bilayer and increased permeability of the fungal membrane.^14^

The region between 1700 and 1500 cm^−1^ encompasses the Amide I and Amide II bands, which are related to secondary protein structures, such as α‐helices and β‐sheets. The spectral analysis revealed a significant increase in absorbance after the application of PDI, especially after the second dose, indicating structural changes in the proteins of *C. albicans*. These changes may be attributed to the oxidation of amino acid residues, denaturation of essential proteins, or enzymatic degradation caused by PDI‐induced oxidative stress. The analysis of the region between 1200 and 900 cm^−1^ revealed significant differences between the spectra of the treated samples and the control, suggesting modifications in the structure of polysaccharides, such as chitin and β‐glucans, which are the main components of the *C. albicans* cell wall. The increase in absorbance in this region after PDI suggests degradation or restructuring of the cell wall, which could compromise fungal integrity and facilitate cell lysis.

Comparing the three experimental groups shows that the second dose of PDI intensified the structural modifications in the hyphae, reinforcing the hypothesis of a cumulative effect of the treatment. The increase in absorbance in critical spectral regions indicates that repeated application of PDI enhances oxidative stress and degradation of cellular components, making *C. albicans* progressively more vulnerable.

## DISCUSSION

Hyphal forms of *C. albicans* exhibited greater sensitivity to AmB than yeast cells, as their growth was inhibited at lower concentrations. The incorporation of PDI further amplified this inhibition, suggesting that PDI could serve as an effective complementary antifungal strategy. A dose‐dependent analysis revealed that two doses of PDI consistently resulted in more potent fungal inhibition across all tested concentrations. This suggests that repeated PDI applications enhance AmB efficacy and provide a more consistent and potent antifungal effect.

A single dose of PDI led to a notable reduction in yeast and hyphal growth. However, the inhibition was more pronounced in yeast cells, suggesting that PDI may be more effective against yeast than hyphal forms. Residual fungal growth persisted, particularly in hyphae, indicating that a single dose may not be sufficient for complete eradication. By contrast, a second PDI dose caused a significantly greater OD reduction, especially in hyphae, supporting the hypothesis that repeated PDI applications are more effective in suppressing hyphal growth, which is often more resistant due to its structural complexity and biofilm formation. The second PDI dose also enhanced AmB activity, leading to a substantial reduction in fungal viability. These findings suggest that multiple PDI doses could improve treatment efficacy, particularly when combined with conventional antifungal agents.

A comparative analysis between yeast and hyphal cells indicates that hyphal forms exhibit higher resistance to treatment with antifungal agents alone. This is consistent with the literature, which suggests that hyphal structures confer increased resilience due to their complex morphology and biofilm‐forming ability. However, PDI with AmB significantly enhances antifungal activity, likely through ROS‐mediated oxidative damage, making PDI a promising adjuvant therapy.

A comparison between yeast and hyphal forms revealed differential responses to treatment. Hyphae demonstrated heightened sensitivity to AmB and PDI, exhibiting a more significant reduction in growth at lower concentrations than yeast cells. This finding suggests that the morphological transition of *C. albicans* influences its susceptibility to treatment, highlighting the importance of targeting hyphal structures in antifungal strategies. At concentrations around 0.26 μg/mL, the combination of PDI—particularly with two doses—significantly enhanced the inhibitory effect of AmB on yeast growth. This concentration emerged as the most effective inducing fungal inhibition, as reflected in the sharp OD decrease in graphical analyses. In the control group, *C. albicans* hyphae without PDI treatment exhibited a significant reduction in growth with increasing concentrations of AmB, confirming its antifungal efficacy. However, the effect was less pronounced in yeast cells, emphasizing the differential susceptibility between the two morphological forms.

The good combination between AmB and PDI has been well documented.^15^ reported that PDI can reduce the MIC required for effective antifungal activity when combined with conventional antifungals. This study corroborates these findings, as the combination of AmB and PDI resulted in a significant OD reduction in yeast and hyphal forms, particularly at intermediate antifungal concentrations. These results support growing evidence that PDI, combined with traditional antifungal agents like AmB, is a powerful strategy for treating *C. albicans* infections. However, *C. albicans* biofilms remain a critical challenge in antifungal therapy due to their dense structure, which limits the penetration of photosensitizers into deeper biofilm layers. This study suggests that multiple PDI applications or combination therapies may be necessary to effectively target mature fungal biofilms, which continue to pose a significant hurdle in *C. albicans* eradication.

The growth curve analysis of *C. albicans* hyphae subjected to PDI revealed a clear dose‐dependent inhibition pattern. In the control group, where no PDI treatment was applied, OD remained the highest, reflecting robust hyphal growth under favorable conditions. This confirms that, without treatment, *C. albicans* can efficiently develop its hyphal structures. A noticeable reduction in OD was observed upon administering a single dose of PDI, indicating that PDI effectively disrupts hyphal growth. However, the inhibition was not as pronounced as achieved with two doses, suggesting that a single application may not be sufficient for complete hyphal suppression. Applying two consecutive PDI doses resulted in a further reduction in OD, demonstrating a cumulative effect. This outcome suggests that repeated PDI exposures may lead to enhanced generation of ROS, thereby intensifying cellular damage and reinforcing fungal inhibition.

The comparison between single and multiple PDI applications highlights the enhanced effectiveness of repeated treatments in targeting *C. albicans* hyphae, which are typically more resistant due to their complex morphology and biofilm‐forming capability. These findings are further supported by Figure 1, which illustrates the dose‐dependent impact of PDI on *C. albicans* hyphal growth. The control group exhibited the highest OD, whereas a single dose of PDI led to a moderate reduction, and two doses resulted in the most significant inhibition. The cumulative effect of multiple PDI applications suggests that enhanced ROS production overwhelms fungal defense mechanisms, ultimately leading to substantial growth suppression.

PDI functions through the generation of ROS, which induces oxidative damage to essential cellular components, including lipids, proteins, and nucleic acids. The greater inhibitory effect observed with two doses suggests that repeated exposures intensify ROS‐mediated cellular stress, making it more difficult for the fungus to repair oxidative damage. Furthermore, the intrinsic resistance of *C. albicans* hyphae, which is attributed to their structural complexity, was effectively countered by multiple PDI doses. This indicates that PDI, mainly when applied in repeated doses, holds promise as an effective antifungal strategy against *C. albicans* infections. Additionally, combining PDI with antifungal agents such as AmB has enhanced fungal inhibition. The better answer between these treatments may result from PDI‐induced membrane damage, facilitating greater antifungal penetration, thereby reducing the required concentration of AmB. This combination strategy is particularly relevant for combating fungal biofilms, which exhibit increased resistance to conventional treatments. The findings underscore the importance of optimizing PDI parameters, such as light dose and photosensitizer concentration, to maximize its antifungal potential. By effectively targeting both yeast and hyphal forms of *C. albicans*, PDI represents a promising therapeutic approach for improving antifungal efficacy, particularly in cases of drug‐resistant infections.

When applied, PDI with the appropriate photosensitizers can effectively inhibit *C. albicans*. The authors emphasized optimizing the light dose and sensitizer concentration to achieve the desired antifungal effect. PDI can generate (ROS), which leads to oxidative damage in fungal cells, resulting in reduced growth and potential cell death.^16^ Ref. 17 discussed the challenges of using PDI alone against biofilms, emphasizing their increased resistance due to the protective extracellular matrix. In agreement with their findings, our results show that a single dose of PDI, while effective in reducing fungal growth, was insufficient for complete inhibition, especially in hyphal cells. However, applying two PDI doses significantly enhanced growth inhibition, suggesting that repeated exposures may be necessary to overcome the structural resistance posed by biofilms and hyphal networks.

The interaction between PDI and conventional antifungal agents has also been previously reported. Amphotericin B, a potent antifungal, targets ergosterol in the fungal cell membrane, leading to membrane destabilization and cell death.^8^ However, *C. albicans* can develop resistance by altering ergosterol content or modifying its binding affinity. Our study corroborates previous reports by demonstrating that the hyphal form of *C. albicans* exhibits greater tolerance to AmB than the yeast form, requiring higher concentrations for effective inhibition. Interestingly, when combined with PDI, the required AmB concentration was significantly reduced, particularly in hyphal cells. This suggests a synergistic interaction, where PDI‐induced oxidative damage compromises the fungal membrane, facilitating increased drug penetration.

The researchers highlighted that biofilm formation is a critical factor in *C. albicans* resistance, as it limits drug penetration and enhances fungal survival. Our results support this observation, as the control hyphal group exhibited significant resistance to AmB alone. However, adding PDI substantially improved the antifungal effect, reducing the minimum inhibitory concentration (MIC) required to inhibit yeast and hyphal growth. This reinforces that PDI can enhance antifungal efficacy by targeting biofilm‐associated resistance mechanisms.^18^

Studies have demonstrated that PDI can compromise the structural integrity of *C. albicans* by degrading key cellular components. The researchers showed that PDI induces the degradation of polysaccharides in the fungal cell wall, weakening its structure. It was reported that ROS generated during PDI can lead to protein denaturation in yeast, as evidenced by changes in FTIR spectra.^19^ Similarly, it was demonstrated that PDI induces lipid peroxidation in yeast, compromising membrane integrity and leading to cell death.^20^ Furthermore, it was highlighted that ROS generated during PDI can modify hydroxyl and amide groups, impairing protein functionality. Additionally, photoinactivation has been shown to oxidize proteins and lipids, directly impacting fungal viability. Researchers reported that PDI alters the structural properties of Candida spp., further underscoring its disruptive effects on fungal cells.^19^

Researchers reported that PDI effectively inhibits *C. albicans*, targeting yeast and hyphal morphologies through ROS‐mediated oxidative damage. Our study aligns with these findings, demonstrating a 75% reduction in yeast growth and an 87.5% reduction in hyphal growth following PDI treatment. Notably, the hyphal form exhibited greater susceptibility to PDI, as evidenced by the substantial decrease in MIC from 1.04 to 0.13 μg/mL when two doses were applied. This represents an 8‐fold reduction in the required AmB concentration, further supporting the potential of PDI as an adjuvant therapy for invasive *C. albicans* infections.^15^, ^21^

The ability of PDI to effectively target both yeast and hyphal forms provides an advantage over conventional antifungal treatments, which often show reduced efficacy against biofilm‐associated infections. The transition from yeast to hyphal form is a critical virulence factor in *C. albicans*, as hyphae are more invasive and resistant to treatment.^21^ However, our results suggest that repeated PDI applications can overcome this resistance by generating sufficient ROS to disrupt fungal structures, making the cells more susceptible to antifungal agents.

Our findings align with previous studies reporting the synergistic effects of PDI when combined with conventional antifungals. Researchers emphasized that optimizing PDI parameters can significantly enhance antifungal efficacy. In our study, the addition of PDI to AmB treatment reduced the MIC from 0.26 to 0.065 μg/mL in yeast cells and from 1.04 to 0.13 μg/mL in hyphal cells, demonstrating that lower AmB concentrations were required when PDI was applied.^16^ This is consistent with researchers who reported that PDI lowers the MIC of antifungal agents, allowing for more effective fungal eradication at reduced drug doses.^22^

These results underscore the therapeutic potential of PDI as an adjunctive strategy, particularly for treating *C. albicans* infections that involve biofilm formation and drug‐resistant strains. Given the challenges associated with fungal resistance to conventional antifungals, the ability of PDI to enhance drug susceptibility presents a promising avenue for future antifungal therapies. Further research should focus on optimizing PDI protocols, including photosensitizer selection, light exposure parameters, and treatment intervals, to maximize its clinical efficacy.

## CONCLUSION

In conclusion, PDI represents a groundbreaking approach in the fight against *C. albicans* infections, offering a promising solution to combat both the yeast and highly resistant hyphal forms of this pathogen. The better effect of PDI, when combined with antifungal agents like AmB, has shown remarkable potential in enhancing treatment efficacy by reducing the necessary antifungal concentrations and significantly inhibiting fungal growth. With its ability to penetrate biofilms and overcome resistance mechanisms associated with hyphal forms, PDI provides a powerful tool for treating persistent and hard‐to‐eradicate infections. FTIR plot analysis suggests that PDI 1 and PDI 2 treatments are causing significant changes in the chemical structure of the yeast, including cell wall damage, protein denaturation, and lipid peroxidation. These results align with the scientific literature, demonstrating photodynamic therapy's effectiveness in controlling pathogenic microorganisms, such as *C. albicans*. By disrupting *Candida albicans*' ability to adapt and survive, PDI opens new doors for innovative, more effective treatments, paving the way for a future where resistant fungal infections can be tackled with greater precision and success.

## Data Availability

The data that supports the findings of this study are available on request to the corresponding author.
